# Supplementary material for: The Dutch Comparative Scale for Assessing Volunteer Motivations among Volunteers and Non-Volunteers: An Adaptation of the Volunteer Functions Inventory
Source: Int J Environ Res Public Health. 2019 Dec 11;16(24):5047. doi: 10.3390/ijerph16245047 (PMC6950329; doi:10.3390/ijerph16245047)
Supplement: Supplementary file 1 [file ijerph-16-05047-s001.pdf]

Supplementary Material Table S1 Volunteer Functions Inventory (Clary et al., 1998)

Please indicate how important or accurate each of the 30 possible reasons for volunteering were for you in doing volunteer work

1. Volunteering can help me to get my foot in the door at a place where I would like to work
2. My friends volunteer
3. I am concerned about those less fortunate than myself
4. People I'm close to want me to volunteer
5. Volunteering makes me feel important
6. People I know share an interest in community service
7. No matter how bad I've been feeling, volunteering helps me to forget about it
8. I am genuinely concerned about the particular group I am serving
9. By volunteering I feel less lonely
10. I can make new contacts that might help my business or career
11. Doing volunteer work relieves me of some of the guilt over being more fortunate than others
12. I can learn more about the cause for which I am working
13. Volunteering increases my self-esteem
14. Volunteering allows me to gain a new perspective on things
15. Volunteering allows me to explore different career options
16. I feel compassion toward people in need
17. Others with whom I am close place a high value on community service
18. Volunteering lets me learn things through direct, hands on experience
19. I feel it is important to help others
20. Volunteering helps me work through my own personal problems
21. Volunteering will help me to succeed in my chosen profession
22. I can do something for a cause that is important to me
23. Volunteering is an important activity to the people I know best
24. Volunteering is a good escape from my own troubles
25. I can learn how to deal with a variety of people
26. Volunteering makes me feel needed
27. Volunteering makes me feel better about myself
28. Volunteering experience will look good on my resume
29. Volunteering is a way to make new friends
30. I can explore my own strengths

1 = not at all important/accurate; 7 = extremely important/accurate

Supplementary Material Table S2 Dutch validated translation of the VFI [14] **(*Dutch 27-item VFI-V)***

Hieronder staan 27 mogelijke redenen voor het doen van vrijwilligerswerk. Wilt u steeds aangeven in hoeverre elk van de genoemde redenen op u van toepassing is?

1. Vrijwilligerswerk kan me helpen een voet tussen de deur te krijgen op een plek waar ik zou willen werken.
2. Mijn vrienden doen vrijwilligerswerk.
3. Ik ben betrokken bij mensen die het minder goed getroffen hebben dan ikzelf.
4. De mensen die dichtbij me staan willen dat ik vrijwilligerswerk doe.
5. Door het doen van vrijwilligerswerk voel ik me belangrijk.
6. Mijn kennissen zijn geïnteresseerd in het leveren van een bijdrage aan de samenleving.
7. Hoe slecht ik me ook voel, vrijwilligerswerk helpt me het te vergeten.
8. Ik ben oprecht betrokken bij de groep die ik help.
9. Door vrijwilligerswerk te doen voel ik me minder eenzaam.
10. Ik kan nieuwe contacten opdoen die mogelijk van pas komen voor mijn bedrijf of carrière.

* *(Door vrijwilligerswerk voel ik me er minder schuldig over dat ik het beter getroffen heb dan anderen.)*[1]

11. Ik kan meer te weten komen over het doel waarvoor ik me inzet.
12. Vrijwilligerswerk verhoogt mijn gevoel van eigenwaarde.
13. Vrijwilligerswerk stelt me in staat dingen in een nieuw perspectief te zien.
14. Vrijwilligerswerk stelt me in staat verschillende carrièremogelijkheden te onderzoeken.
15. Ik leef mee met mensen die hulp nodig hebben.
16. Mensen die dichtbij me staan hechten veel waarde aan het leveren van een bijdrage aan de samenleving.
17. Door vrijwilligerswerk kan ik dingen leren door directe, praktische ervaring op te doen.
18. Ik vind het belangrijk anderen te helpen.
19. Vrijwilligerswerk helpt me mijn eigen problemen te verwerken.
20. Vrijwilligerswerk zal me helpen succesvol te zijn in mijn beroep.

* *(Ik kan iets doen voor een doel dat belangrijk voor me is.)*[2]

21. Vrijwilligerswerk is een belangrijke bezigheid voor de mensen die ik het beste ken.
22. Vrijwilligerswerk is een goede afleiding van mijn eigen problemen.
23. Ik kan leren omgaan met verschillende soorten mensen.
24. Vrijwilligerswerk geeft me het gevoel dat ik nodig ben.
25. Vrijwilligerswerk geeft me een beter gevoel over mezelf.
26. Ervaring met vrijwilligerswerk staat goed op mijn cv.

* *(Vrijwilligerswerk is een manier om nieuwe vrienden te maken.)*[3]

27. Ik kan mijn eigen sterke punten verkennen.

1 = helemaal niet van toepassing / 7 = heel erg van toepassing.

---

[1] This item has been deleted in the validation process and is therefore not part of the Dutch version of the VFI. Originally, this item is stated as Item K (‘Doing volunteer work relieves me of some of the guilt over being more fortunate than others’

[2] This item has been deleted in the validation process and is therefore not part of the Dutch version of the VFI. Originally, this item is stated as item V (‘I can do something for a cause that is important to me’)

[3] This item has been deleted in the validation process and is therefore not part of the Dutch version of the VFI. Originally, this item is stated as item CC (‘Volunteering is a way to make new friends’)

Supplementary Material Table S3 Adaptation of the Dutch translation of the original VFI, applicable to non-volunteering individuals (containing 27 items, corresponding to the items included in the validated Dutch VFI)

Hieronder staan 27 mogelijke redenen voor het doen van vrijwilligerswerk. Wat zouden voor u redenen zijn om wel vrijwilligerswerk te gaan doen?

1. Vrijwilligerswerk zou me helpen een voet tussen de deur te krijgen op een plek waar ik zou willen werken.
2. Mijn vrienden doen vrijwilligerswerk.
3. Ik ben betrokken bij mensen die het minder goed getroffen hebben dan ikzelf.
4. De mensen die dichtbij me staan willen dat ik vrijwilligerswerk doe.
5. Door het doen van vrijwilligerswerk zou ik me belangrijk voelen.
6. Mijn kennissen zijn geïnteresseerd in het leveren van een bijdrage aan de samenleving.
7. Hoe slecht ik me ook voel, vrijwilligerswerk zou me helpen het te vergeten.
8. Ik ben oprecht betrokken bij de groep die ik zou helpen.
9. Door vrijwilligerswerk te doen zou ik me minder eenzaam voelen.
10. Ik zou nieuwe contacten opdoen die mogelijk van pas komen voor mijn bedrijf of carrière.
11. Ik kan meer te weten komen over het doel waarvoor ik me zou inzetten.
12. Vrijwilligerswerk zou mijn gevoel van eigenwaarde verhogen.
13. Vrijwilligerswerk zou me in staat stellen dingen in een nieuw perspectief te zien.
14. Vrijwilligerswerk zou me in staat stellen verschillende carrièremogelijkheden te onderzoeken.
15. Ik leef mee met mensen die hulp nodig hebben.
16. Mensen die dichtbij me staan hechten veel waarde aan het leveren van een bijdrage aan de samenleving.
17. Door vrijwilligerswerk zou ik dingen leren door directe, praktische ervaring op te doen.
18. Ik vind het belangrijk anderen te helpen.
19. Vrijwilligerswerk zou me helpen mijn eigen problemen te verwerken.
20. Vrijwilligerswerk zou me helpen succesvol te zijn in mijn beroep.
21. Vrijwilligerswerk is een belangrijke bezigheid voor de mensen die ik het beste ken.
22. Vrijwilligerswerk zou een goede afleiding van mijn eigen problemen zijn.
23. Ik zou leren omgaan met verschillende soorten mensen.
24. Vrijwilligerswerk zou me het gevoel geven dat ik nodig ben.
25. Vrijwilligerswerk zou me een beter gevoel over mezelf geven.
26. Ervaring met vrijwilligerswerk zou goed staan op mijn cv.
27. Vrijwilligerswerk is een manier om nieuwe vrienden te maken.

1 = helemaal niet van toepassing / 7 = heel erg van toepassing.

Supplementary Material Table S4 Pattern Matrix Volunteer Sample (*N* = 4,208) (Principal-Axis Factor Analysis, Oblique Rotation, Six factors pre-specified)

Dutch 27-item VFI-V **(6 factors, 27 items)**	**Factor**					
	**1**	**2**	**3**	**4**	**5**	**6**
**1. Understanding**						
12. Ik kan meer te weten komen over het doel waarvoor ik me inzet	.446					
14. Vrijwilligerswerk stelt me in staat dingen in een nieuw perspectief te zien	.558					
18. Door vrijwilligerswerk kan ik dingen leren door directe, praktische ervaring op te doen	.661					
25. Ik kan leren omgaan met verschillende soorten mensen	.519					
30. Ik kan mijn eigen sterke punten verkennen	.721					
**2. Career**						
1. Vrijwilligerswerk kan me helpen een voet tussen de deur te krijgen op een plek waar ik zou willen werken		-.635				
10. Ik kan nieuwe contacten opdoen die mogelijk van pas komen voor mijn bedrijf of carrière		-.703				
15. Vrijwilligerswerk stelt me in staat dingen in een nieuw perspectief te zien						
21. Vrijwilligerswerk zal me helpen succesvol te zijn in mijn beroep		-.822				
28. Ervaring met vrijwilligerswerk staat goed op mijn cv.		-.646				
		-.618				
**3. Values**						
3. Ik ben betrokken bij mensen die het minder goed getroffen hebben dan ikzelf			.575			
8. Ik ben oprecht betrokken bij de groep die ik help			.551			
16. Ik leef mee met mensen die hulp nodig hebben			.867			
19. Ik vind het belangrijk anderen te helpen			.787			
**4. Protective**						
7. Hoe slecht ik me ook voel, vrijwilligerswerk helpt me het te vergeten				.370		
9. Door vrijwilligerswerk te doen voel ik me minder eenzaam				.451		
20. Vrijwilligerswerk helpt me mijn eigen problemen te verwerken				.823		
24. Vrijwilligerswerk is een goede afleiding van mijn eigen problemen				.837		
**5. Social**						
2. Mijn vrienden doen vrijwilligerswerk					-.592	
4. De mensen die dichtbij me staan willen dat ik vrijwilligerswerk doe					-.483	
6. Mijn kennissen zijn geïnteresseerd in het leveren van een bijdrage aan de samenleving					-.730	
17. Mensen die dichtbij me staan hechten veel waarde aan het leveren van een bijdrage aan de					-.595	

samenleving						
23. Vrijwilligerswerk is een belangrijke bezigheid voor de mensen die ik het beste ken					-.499	
**6. Enhancement**						
5. Door het doen van vrijwilligerswerk voel ik me belangrijk						.610
13. Vrijwilligerswerk verhoogt mijn gevoel van eigenwaarde						.676
26. Vrijwilligerswerk geeft me het gevoel dat ik nodig ben						.475
27. Vrijwilligerswerk geeft me een beter gevoel over mezelf						.574

Supplementary Material Table S5 Pattern Matrix Non-Volunteer Sample (N =3,404) (Principal-Axis Factor Analysis, Oblique Rotation, Six factors pre-specified)

**Non-volunteer items (6 factors, 27 items)**	**Factor**					
	**1**	**2**	**3**	**4**	**5**	**6**
**1. Understanding**						
12. Ik kan meer te weten komen over het doel waarvoor ik me zou inzetten	-.374	.323				
14. Vrijwilligerswerk zou me in staat stellen dingen in een nieuw perspectief te zien	-.423					.361
18. Door vrijwilligerswerk zou ik dingen leren door directe, praktische ervaring op te doen	-.565					
25. Ik zou leren omgaan met verschillende soorten mensen	-.630					
30. Ik zou mijn eigen sterke punten verkennen	-.639					
**2. Career**						
1. Vrijwilligerswerk zou me helpen een voet tussen de deur te krijgen op een plek waar ik zou willen werken		.628				
10. Ik zou nieuwe contacten opdoen die mogelijk van pas komen voor mijn bedrijf of carrière		.714				
15. Vrijwilligerswerk zou me in staat stellen dingen in een nieuw perspectief te zien						
21. Vrijwilligerswerk zou me helpen succesvol te zijn in mijn beroep		.864				
28. Ervaring met vrijwilligerswerk zou goed staan op mijn cv.		.530		.301		
		.638				
**3. Values**						
3. Ik ben betrokken bij mensen die het minder goed getroffen hebben dan ikzelf			.575			
8. Ik ben oprecht betrokken bij de groep die ik zou helpen			.508			
16. Ik leef mee met mensen die hulp nodig hebben			.889			
19. Ik vind het belangrijk anderen te helpen			.832			
**4. Protective**						
7. Hoe slecht ik me ook voel, vrijwilligerswerk zou me helpen het te vergeten				.535		
9. Door vrijwilligerswerk te doen zou ik me minder eenzaam voelen				.623		
20. Vrijwilligerswerk zou me helpen mijn eigen problemen te verwerken				.681		
24. Vrijwilligerswerk zou een goede afleiding van mijn eigen problemen zijn				.743		
**5. Social**						
2. Mijn vrienden doen vrijwilligerswerk					.642	
4. De mensen die dichtbij me staan willen dat ik vrijwilligerswerk doe					.433	
6. Mijn kennissen zijn geïnteresseerd in het leveren van een bijdrage aan de samenleving					.776	

17. Mensen die dichtbij me staan hechten veel waarde aan het leveren van een bijdrage aan de samenleving			.354		.519	
23. Vrijwilligerswerk is een belangrijke bezigheid voor de mensen die ik het beste ken					.499	
**6. Enhancement**						
5. Door het doen van vrijwilligerswerk zou ik me belangrijk voelen				.523		
13. Vrijwilligerswerk zou mijn gevoel van eigenwaarde verhogen				.578		
26. Vrijwilligerswerk zou me het gevoel geven dat ik nodig ben	-.479			.485		
27. Vrijwilligerswerk zou me een beter gevoel over mezelf geven	-.341			.646		

Supplementary Material Table S6 Eliminated items - discussion of potential reasons for incomparability between the volunteer and non-volunteer samples

Items	Reason for elimination of item	Potential explanation for incomparability between volunteer and non-volunteer samples
**1. Understanding**		
12. I can learn more about the cause for which I am working	Relatively low factor loading on intended factor in both groups AND cross-loading on factor *Career* in non-volunteer sample AND interpretation of this item could be difficult for non-volunteers because 'the cause' to work for is not specified for non-volunteers.	The interpretation of this item is probably difficult for non-volunteering individuals, because 'the cause' to work for is not specified for non-volunteers. The cross-loading on the factor *Career* in the non-volunteer group seems to reflect that non-volunteering individuals assess this item more from a paid work perspective.
14. Volunteering allows me to gain a new perspective on things	Low factor loading on intended factor in non-volunteer sample AND cross-loading on a factor without any other items.	This item seems to be difficult to interpret for non-volunteering individuals, probably especially for those without any previous volunteer experience. For this specific group, it may be very hard to imagine the benefits of volunteering, especially more abstract benefits like the one described in this item.
**2. Career**		
21. Volunteering will help me to succeed in my chosen profession	Cross-loading on factor *Protective* in non-volunteer sample.	While this items works well among the volunteer sample, it seems to work less good among non-volunteers. Especially for non-volunteers without any previous volunteering experience, it could be difficult to imagine benefits of volunteering on other activities in life, such as paid work.
**3. Values**		
8. I am genuinely concerned about the particular group I am serving	Relatively low factor loading on intended factor in both groups AND interpretation of this item could be difficult for non-volunteers because 'the particular group' to serve is not specified for non-volunteers.	The interpretation of this item is probably difficult for non-volunteering individuals, because 'the particular group' to serve is not specified for non-volunteers and it could be hard for them to imagine the group they would serve when opting for volunteering.

**4. Protective**<br>7. No matter how bad I've been feeling, volunteering helps me forget about it<br>9. By volunteering I feel less lonely	Both items had a relatively low factor loading on the intended factor in both groups AND factor loadings in non-volunteer sample were substantially lower than in volunteer sample.	These items may be difficult to assess by individuals who did not feel bad or lonely, probably causing the relatively low factor loadings in both groups. Moreover, for non-volunteering individuals, if they have been feeling bad or lonely, it may be very difficult to imagine whether and to what extent volunteering would help in eliminating negative feelings.
**5. Social**<br>4. People I'm close to want me to volunteer	Relatively low factor loading on intended factor in both groups.	This item is quite different from the other four items belonging to the *Social* factor. The other four items all assess the importance of contributing to society for people in the social network of the respondent. Item 4, in contrast, assesses whether these people in the social network of the respondent transfer their opinion about the importance of societal contribution to the respondent, by desiring the respondent to contribute as well.
**6. Enhancement**<br>26. Volunteering makes me feel needed<br>27. Volunteering makes me feel better about myself	Both items had a cross-loading on the factor *Understanding* in the non-volunteer sample.	For non-volunteering individuals, it could be difficult to imagine the potential positive influence on one's self-esteem. This could moreover be different for non-volunteers who have never volunteered, as compared to those with volunteering experience, who may be able to imagine benefits of volunteering.
